# Synergistic Enhanced Thermal Conductivity and Crack Resistance of Reactor Epoxy Insulation with Boron Nitride Nanosheets and Multiwalled Carbon Nanotubes

**DOI:** 10.3390/nano12183235

**Published:** 2022-09-18

**Authors:** Jun Yang, Zhijie Chen, Longyi Liang, Zhiwen Guan, Junwen Ren

**Affiliations:** College of Electrical Engineering, Sichuan University, Chengdu 610065, China

**Keywords:** epoxy, boron nitride nanosheets, carbon nanotubes, thermal conductivity, epoxy crack

## Abstract

Epoxy composites with high thermal conductivity, excellent dielectric, and mechanical properties are very promising for solving epoxy cracking faults in reactors and for extending their service life. In this work, we report on epoxy composites enhanced by ternary fillers of boron nitride nanosheets (BNNSs), multiwalled carbon nanotubes (MWCNTs), and silica (SiO_2_) nanoparticles. The obtained BNNSs/MWCNTs/SiO_2_/epoxy composites exhibit a high thermal conductivity of 0.9327 W m^−1^ K^−1^, which is more than 4-fold higher than that of pure epoxy. In addition, the resultant composites present an improved mechanical strength (from 2.7% of epoxy to 3.47% of composites), low dielectric constant (4.6), and low dielectric loss (0.02). It is believed that the integration of multifunctional properties into epoxy composites provides guidance for optimizing the design of high-performance materials.

## 1. Introduction

With the rapid increase in electricity consumption, the requirements for grid stability and power quality are increasing with each passing day. Various reactors widely installed in the grid represent critical equipment for suppressing grid fluctuations and maintaining bus fault voltage. In the ultrahigh–voltage DC transmission project, saturable reactors play an essential role in the standard opening and closing of the converter valve [1,2,3,4,5,6]. At present, epoxy resin is usually used in the insulation design of reactors to meet insulation requirements. However, the epoxy resin insulation layer undergoes aging and cracking during the use process, and insulation failure occurs. At the same time, epoxy resin is also widely used in other types of power equipment as insulation or support material. Therefore, extending the service life of the epoxy resin insulation layer and reducing its cracking and damage can significantly improve the stability and safety of power grid operation and can significantly reduce the accident rate.

Currently, the increment in the voltage level of the power grid, the transmission capacity of each outlet circuit, and the grid–connected capacity of green electricity such as photovoltaic or wind power may lead to an increase in the degree of fluctuation of the power grid. Therefore, the impulse voltage in each reactor increases, bringing about an increase in losses, especially hysteresis losses in its core and dielectric losses in the dielectric insulation [7]. The lost electric energy convert into heat, which increases the temperature of the insulation layer. In the case of a high inrush current, the coil induces a large force due to electromagnetic induction, which will squeeze the epoxy insulation; simultaneously, the temperature of the contact part between the epoxy resin and the core increases rapidly. Both effects accelerate the aging of the epoxy insulation layer and weaken its performance in mechanical and electrical aspects [8,9,10].

Among the main focuses of research to solve the problem of aging, cracking, and life extension of epoxy resin insulation is to improve the thermal conductivity of the insulation layer and, thus, reduce the mechanical damage caused by lopsided thermal expansion [1,11,12,13]. In addition, epoxy with higher thermal conductivity is beneficial for rapidly cooling the hotspot temperature of devices, and relieving the thermal stress concentration caused by local overheating. The lower temperature can slow down the aging rate of the epoxy resin insulation layer, which is more beneficial to extend the service life of reactors [14]. Zhao et al. [15] modified epoxy through mixing 2D BNNSs and 1D boron nitride microspheres to construct multidimensional thermal conductivity channels, thus enhancing the heat path density. The thermal conductivity was 5-fold higher than that of the pure epoxy at 30 wt.% filler loading. In addition, Chen et al. [16] synthesized a connected boron nitride nanosheet aerogel supported using a nanocellulose backbone. The thermal conductivity of the obtained material was enhanced by a factor of 1400% when the filler loading was 9.6 vol.%.

The improvement of the mechanical properties also displays a significant effect on the aging and cracking of materials. Godara et al. [17] mixed different functionalized carbon nanotubes (CNTs) into epoxy resin matrix, contributing to a 32% reduction in the thermal expansion coefficient of the composites. In addition, using CNTs as a filler, the yield strength of the epoxy resin composites was increased by 80%. Chen et al. [18] claimed that the flexural strength of epoxy composites could be significantly improved by adding only 0.75% CNTs.

In addition, previous researches have demonstrated that dielectric properties of materials can be optimized by incorporating a certain amount of silicon dioxide (SiO_2_) nanoparticles [19,20,21,22]. Therefore, it is believed that a combination of different types of fillers can be used to prepare multifunctional composites to meet specific needs. 

Here, we report a method to prepare highly thermally conductive and anti-cracking epoxy composites using a combination of BNNS, MWCNTs, and SiO_2_ as ternary fillers. The thermal, mechanical, and dielectric properties of the obtained BNNSs/MWCNTs /SiO_2_/epoxy composites have been systematically investigated. It is showed that composites simultaneously exhibit an improved mechanical strength and high thermal conductivity without compromising of the excellent dielectric performance of epoxy matrix. We believe that the composites fabricated in this work shed a new light on solving the aging and cracking problem of the epoxy insulation layer of the reactor.

## 2. Materials and Methods

### 2.1. Materials

h-BN with an average particle size of 20 μm was provided by Aladdin Co., Ltd., Shanghai, China. Bisphenol A glycidyl ether (E-51 epoxy) with an epoxide equivalent weight of 182–192, C12–14 alkyl glycidyl ether (epoxy thinner AGE), and dimethylaminomethylphenol (epoxy catalyst DMP-30) were provided by Nantong Xingchen Synthetic Materials Co., Ltd., Nantong, China. Methyltetrahydrophthalic anhydride (epoxy curing agent MTHPA) was purchased from Henan Huineng Resin Co., Ltd., Henan, China. Aminated multiwalled carbon nanotubes were purchased from Kaisa (Huizhou, China) New Materials Co., Ltd. (Guangzhou, China). Nano-silica nanoparticles were produced and provided by Zhuotai New Material Technology Co., Ltd. (Tangshan, China). Bis-amino functional silane (silane coupling agent KH-560) was provided by Aladdin Co., Ltd. (Beijing, China). Other materials were purchased from Chengdu Kelon Chemical Co., Ltd. (Chengdu, China) and were used as instructed, such as isopropyl alcohol, deionized water, and anhydrous ethanol.

### 2.2. Liquid-Phase Stripping of BNNSs

**Figure 1:**
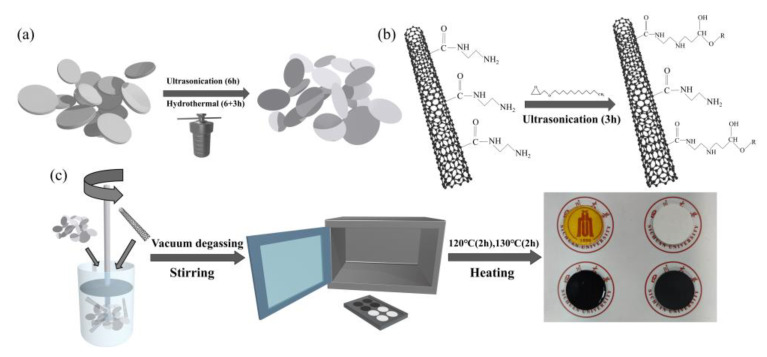
(**a**) Liquid-phase exfoliation process of h-BN; (**b**) surface functionalization of MWCNTs; (**c**) fabricating procedure of BNNSs/MWCNTs /SiO_2_/epoxy composites.

BNNSs were prepared via liquid-phase exfoliation of h-BN using salt ions in combination with isopropanol, using extended water bath sonication and a hydrothermal reaction. The process is described in Figure 1a. Specifically, the exfoliation method was as described in [15,23]. Briefly, 1 g of lithium citrate and 1 g of 20 μm h-BN were added to 100 mL of a mixture of isopropanol and water (3:1) and then placed in an ultrasonic probe (Ningbo Scientz Biotechnology Co., Ltd., Ningbo, China) for 20 min. The well-dispersed mixture was treated with 40 kHz ultrasound (Ningbo Scientz Biotechnology Co., Ltd., Ningbo, China) for 3 h and then removed, before repeating the above process once. The obtained dispersion was removed and transferred to a stainless-steel autoclave lined with tetrafluoroethylene, heated, and hydrothermally treated at 180 °C for 12 h. After cooling, the dispersion was taken out and washed three times with deionized water by suction filtration (Tianjin Jinteng Experimental Equipment Co., Ltd., Tianjin, China) to obtain BNNSs dispersed in deionized water. The dispersion was hydrothermally treated at 180 °C for 6 h, and then removed and filtered to obtain a white powder solid. Finally, the obtained white powder solid was dried in a vacuum environment at 60 °C for 48 h, yielding the stripped BNNSs.

### 2.3. Preparation of Nano-SiO_2_@KH560 Nanoparticles and MWCNTs@AGE

In order to achieve better bonding between the nano-SiO_2_ nanoparticles and epoxy resin substrate, surface functionalization was needed. Accordingly, the nano-SiO_2_ nanoparticles were treated by hydrolysis of the silane coupling agent KH-560 (3–Glycidoxypropyltrimethoxysilane) so that the hydroxyl group on the surface of the nano-SiO_2_ nanoparticles and the hydroxyl group of the coupling agent formed hydrogen bonds before dehydrating to form Si–O bonds. Then, the epoxy groups on the silane coupling agent were grafted to the surface of the nano-SiO_2_ nanoparticles. The preparation process was as follows: 5 g of SiO_2_ nanoparticles was placed in 100 g of anhydrous ethanol, stirred at 500 rpm, and treated in an ultrasonic water bath for 1 h to make the dispersion uniform. Then, 5 g of KH-560 was added to the dispersion, and the water bath was further heated and sonicated for 3 h. The obtained dispersion was allowed to stand for 24 h, and then filtered and washed three times using acetone to remove the residual silane coupling agent. Finally, the obtained white solid was dried in a vacuum oven at 60 °C for 24 h to prepare the obtained SiO_2_@KH560 nanoparticles, which are hereafter referred to as nano-SiO_2_ nanoparticles.

Subsequently, 1 g of MWCNTs–NH_2_ was homogeneously mixed with 20 g of AGE, and then the mixture was sonicated in a water bath at 40 kHz (200 W) for 4 h. After removal, the mixture was left to stand at room temperature for 24 h to ensure the full reaction between AGE and nanotubes, as described in Figure 1b. The solvent was removed using a filter extractor and then washed several times with acetone to remove the residual AGE on the surface. Finally, the solid obtained was dried in a vacuum environment at 80 °C for 24 h and ground to prepare MWCNTs@AGE.

### 2.4. Preparation of BNNSs/MWCNTs@AGE/SiO_2_@KH560/Epoxy Composites

The composites were prepared using a simple scheme of solid–liquid blending and heat curing. First, SiO_2_@KH560, BNNSs, and MWCNTs@AGE were weighed to the calculated masses according to the corresponding filler ratios and dissolved into 10 mL of acetone solvent and ultrasonically dispersed using 40 kHz (200 W) for 10 min to obtain a homogeneous mixed liquid. Then, epoxy resin (E-51), epoxy resin diluent (AGE), hardener, and catalyst were added to the dispersion in the weight ratio of 40:10:40:1. The epoxy resin and AGE were added first and stirred at 400 rpm for 1 h at room temperature; then, the hardener and catalyst were added before continuing to stir for 1 h to uniformly mix the epoxy resin, hardener, and filler particles. Finally, stirring at 1500 rpm for 10 min was required to remove most of the acetone. The mixture was then placed into a vacuum oven at 60 °C, evacuated, and allowed to degas for 1 h to remove the remaining acetone and the large number of air bubbles that entered the epoxy resin mixture during the mixing process. Finally, the mixture was poured into a preheated mold at 60 °C, heated to 80 °C, and degassed by vacuum for 1 h to remove the remainder of the acetone and air bubbles. Then, the composite was cured at 120 °C for 2 h under atmospheric pressure and then heated to 130 °C for 2 h. After it cooled naturally, it was taken out of the mold and sanded flat using sandpaper to obtain the desired specimen. The preparation process of the composite material is shown in Figure 1c.

### 2.5. Characterization

The microstructure and morphology of BNNSs, MWCNTs, h-BN, and composites were characterized using scanning electron microscopy (SEM, 5 kV, ZEISS, Gemini 400, Oberkochen, BW, Germany). The dielectric response and insulation properties of the composites were characterized in the frequency range 10^1^–10^6^ using a broadband dielectric impedance relaxation spectrometer Concept 50 (Novocontrol Technologies, Montabaur, RP, Germany). The samples were sprayed with gold before testing. The thermal conductivity of the composites was tested at room temperature (25 °C) using the planar heat flow method (DRL-III, Tan Instrument Co., Xiangtan, China). The X-ray photoelectron spectra (XPS) of the samples from 0 to 1200 eV were obtained using an EscaLab 250Xi (Thermo Fisher, Waltham, MA, USA). Fourier–transform infrared spectra (FT–IR) were recorded for each sample from 500 to 4000 cm^−1^ using a Nicolet 460 spectrometer (Thermo Fisher, Waltham, MA, USA). The Raman value spectra were obtained in the range of 100–2000 cm^−1^ using a Renishaw 2000 Raman system (Renishaw, London, UK) with 633 nm laser excitation. The thermogravimetric analysis of the samples was performed using a NETZSCH STA449C, and the test conditions were a nitrogen atmosphere in the temperature range 40–600 °C, at a heating rate of 5 °C/min. A Discovery DMA850 (TA Instruments, New Castle, DE, USA) was used to test the dynamic thermodynamic properties of the samples from 30 to 230 °C under the condition of a single cantilever fixture. 

## 3. Results and Discussion

**Figure 2:**
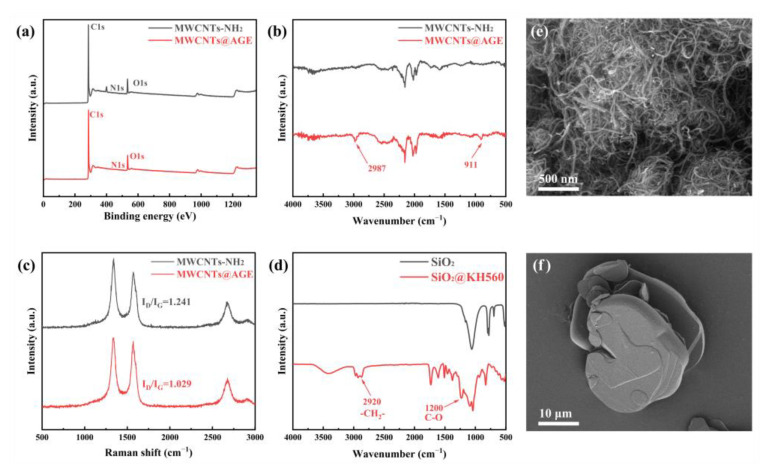
Characterization of SiO_2_, SiO_2_@KH560, MWCNTs, and MWCNTs@AGE. (**a**–**c**) XPS general spectra, FT-IR spectra, and Raman spectra of MWCNTs and MWCNTs@AGE; (**d**) FT-IR spectra of SiO_2_ and SiO_2_@KH560; (**e**,**f**) SEM images of MWCNTs and h-BN.

**Figure 3:**
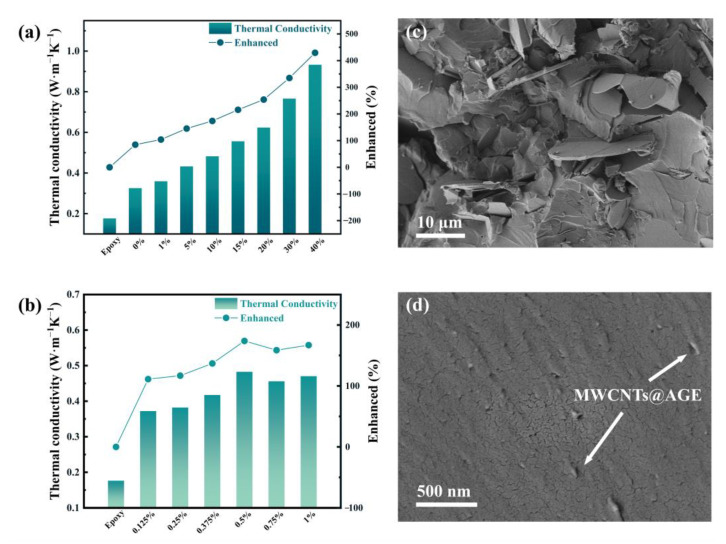
(**a**) Thermal conductivity and enhanced efficiency of composites with BNNSs (with 0.5 wt.% MWCNTs@AGE). (**b**) Thermal conductivity and enhanced efficiency of composites with MWCNTs@AGE (with 10 wt.% BNNSs). (**c**,**d**) SEM images of BNNSs/MWCNTs/SiO_2_/epoxy composites.

Figure 2e shows the SEM image of MWCNTs–NH_2_, which can be seen to be agglomerated under natural conditions. Due to the strong van der Waals force interaction between MWCNTs–NH_2_, a certain duration of ultrasonic treatment is required to untwist the entangled nanotubes when used. We used XPS analysis to demonstrate whether the functionalized groups were successfully grafted onto the nanotube surface (Figure 2a). The C1s peak, N1s peak, and O1s peak in the surface elemental analysis spectra of MWCNTs–NH_2_ and MWCNTs@AGE were compared. It can be found that the intensity of the N1s peak located at 400 eV was significantly weakened, clearly indicating that the AGE molecule was successfully grafted to the surface of MWCNTs–NH_2_. Furthermore, the intensity of the peaks of other elements did not change significantly, indicating that the removal of the amino group had some effect on the grafting, while the surface structure of the nanotubes was not greatly affected. In addition, Figure 2b shows the FT-IR spectra of MWCNTs–NH_2_ and MWCNTs@AGE. Their comparison revealed that the stretching vibration peak of the saturated C–H bond appeared more strongly at 2987 cm^−1^ for MWCNTs@AGE compared to MWCNTs–NH_2_, highlighting the long link of AGE attached to its surface, while the stretching vibration peak of the epoxy group at 910 cm^−1^ could also be observed in its spectrum. This also indicates that the AGE molecule was indeed grafted onto the surface of MWCNTs–NH_2_. FT–IR was also used to characterize the changes in the nano-SiO_2_ nanoparticles before and after modification (Figure 2d). The modified nano-SiO_2_ nanoparticles showed multiple new peaks in the range of 2920 to 2850 cm^−1^. The weak absorption peaks are the antisymmetric and symmetric stretching vibration peaks of −CH2−, respectively. The new peak at 1200 cm^−1^ represents a vibrational peak generated by the C–O bonds brought in by the coupling agent. Several other main peaks were present indicating the transverse, longitudinal, symmetric, and bending vibrational peaks of the Si–O–Si bond. These all indicate that there are organic segments on the surface of the modified nano-SiO_2_. The Raman spectral test results of MWCNTs–NH_2_ and MWCNTs@AGE are shown in Figure 2c. The ratio of the spectral intensity in the D-band (~1300 cm^−1^) and G-band (~1580 cm^−1^), i.e., the ratio of I_D_ to I_G_, changed, which was related to the hybridization of carbon atoms in MWCNTs and on the surface after grafting AGE groups, indicating that our grafting was indeed successful. As shown in Figure 1a, BNNSs are produced by treating h-BN through ultrasonic stripping and a hydrothermal reaction. Due to the high temperature and pressure of the hydrothermal reaction and the added lithium ions, the interlayer force of h-BN was weakened, and the interlayer distance increased before completely separating. As a result, the thick, multilayered h-BN (Figure 2f) was stripped into thin, single–layered BNNSs (Figure 3c).

Since SiO_2_ nanoparticles can improve the dielectric constant, 0.5% SiO_2_ is added for optimization; all studies in this paper are based on the addition of 0.5% silica. Figure 3a,b show the variation of the thermal conductivity of the composites with the content of MWCNTs@AGE and BNNSs. Thanks to the higher thermal conductivity of the BNNS, the thermal conductivity of the composites increased substantially with the increase in filler content of MWCNTs@AGE and BNNSs. It is important to note that the growth rate of thermal conductivity at a BNNSs filler ratio below 10% was significantly lower than that above 10%. This trend can be clearly reflected in the change of the slope in the graph, whereby the first half was significantly less steep than the second half. When the proportion of BNNSs filler reached 40 wt.%, and when 0.5% SiO_2_ and 0.5% MWCNTs@AGE were added, the thermal conductivity of the composites increased significantly, reaching 0.9372 W m^−1^ K^−1^, i.e., a 529% increase in thermal conductivity compared to the epoxy (0.1762 W m^−1^ K^−1^). The trend of thermal conductivity with the filler gradient design was consistent, which can be explained by the isolated island effect in the filler modification of polymeric materials. The phonon spectra of polymer materials and BNNSs are different, resulting in serious phonon scattering at the interface; thus, there is a high interface thermal resistance between the two materials [24,25,26]. Therefore, it is difficult to form efficient thermal conductivity paths between BNNSs and low-conductivity fillers. However, as the filler ratio increases, the average distance between BNNSs shrinks, which shortens the heat path through the epoxy. Accordingly, it is easier to overlap between the fillers to form efficient phonon channels. Therefore, a higher filler ratio of BNNSs leads to a higher thermal conductivity of the composite, which improves the rate of thermal conductivity enhancement.

A large amount of fillers will affect the production and processing of polymers. Considering the comprehensive properties such as thermal conductivity, we add 10% BNNS to the composite material. Furthermore, Figure 3b represents the effect of the filler ratio of MWCNTs@AGE on the thermal conductivity of the composites. It should be noted that all the MWCNTs@AGE samples in this paper contained 10 wt.% BNNSs and 0.5 wt.% SiO_2_. Therefore, the 0.125 wt.% filler ratio shown in Figure 3b achieved more than 100% improvement compared to the pure epoxy resin, which was not simply due to the improvement in thermal conductivity brought by MWCNTs@AGE.

When the filler ratio of MWCNTs@AGE reached 0.5 wt.%, the thermal conductivity was 0.4824 W m^−1^ K^−1^ compared to 0.3721 W m^−1^ K^−1^ at the 0.125 wt.% filler ratio, representing an improvement of ~30%. However, as seen in Figure 3b, there was a trend of the thermal conductivity increasing and then decreasing with the increase in filler ratio. At ≤0.5 wt.% filler ratio, the thermal conductivity tended to be positively correlated with the filler ratio, mainly due to the extremely high thermal conductivity of MWCNTs (~3000 W m^−1^ K^−1^). MWCNTs had a high aspect ratio and formed interconnected thermal conductivity channels inside the epoxy resin, which could significantly improve the overall thermal conductivity of the composite. The thick resin spacing between BNNSs fillers and the high interfacial thermal resistance of both elements were mentioned previously, which would hinder the formation of thermal conductivity channels. In contrast, smaller-scale SiO_2_ nanoparticles and MWCNTs@AGE could be directly lapped onto BNNSs, bypassing the interfacial thermal resistance with the substrate and forming thermal conductivity channels directly between the isolated BNNSs, which allowed more accessible and multidimensional phonon transport and, thus, uniform heat diffusion within the composite. When the filler content reached a certain ratio, the epoxy substrate could establish a “multidimensional thermal conductivity highway” with high heat transfer efficiency. However, compared with BNNSs, MWCNTs have a significantly higher surface tension and interfacial thermal resistance between nanotubes and substrates [27,28,29,30], making it more challenging to adhere MWCNTs to polymer substrates [31,32,33]. Moreover, the phonon spectra of MWCNTs and polymers differ significantly. These factors lead to a significant scattering effect of the interface on phonon conduction, which is not conducive to improving the thermal conductivity of the material [34,35].

**Figure 4:**
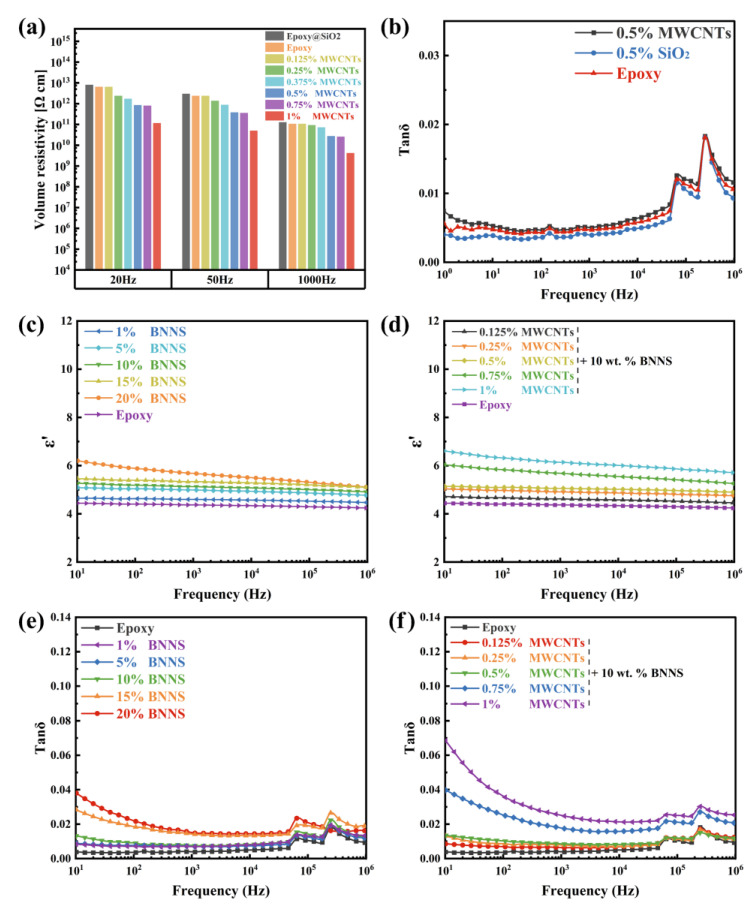
(**a**) Resistivity of composites with different contents of MWCNTs@AGE (All samples contain SiO_2_); (**b**) dielectric loss of pure epoxy, epoxy/SiO_2_@KH560 and epoxy/MWCNTs; (**c**,**d**) dielectric constant of composites with BNNSs and MWCNTs@AGE; (**e**,**f**) dielectric loss of composites with different contents of BNNSs and MWCNTs@AGE (the samples with different concentrations of MWCNTs contain the same amount of BNNSs. All samples contain SiO_2_).

After the surface modification of MWCNTs–NH_2_ with the epoxy diluent AGE, some of the amino groups on the surface of the nanotubes reacted with the ether bonds in AGE, confirming that the epoxy groups were adhered onto the surface of the nanotubes. The epoxy curing process produced epoxy groups on the nanotube surface, which could also participate in the reaction. Such covalent bonding could narrow the distance between MWCNTs@AGE and epoxy resin substrate, promote the phonon coupling between the two phases in the heat transfer process, reduce the contact thermal resistance between the MWCNTs@AGE and epoxy resin substrate bonding surface, and improve the thermal conductivity of the composite material. Furthermore, it also improved the crosslink density of epoxy resin, which was conducive to the improvement of the mechanical properties of the composites. Moreover, large van der Waals forces between MWCNTs facilitate their agglomeration, and the introduction of some groups via surface functionalization treatment can improve their dispersion in the polymer matrix [36,37,38]. However, according to the test results, when the filler ratio of MWCNTs@AGE was higher than 0.5 wt.%, the thermal conductivity seemingly decreased instead. This may be because the nanotube proportion was too high, leading to severe agglomeration, preventing a uniform distribution inside the material and, thus, cooperation with the other fillers to form more dispersed and efficient thermal conductivity channels. Additionally, the agglomerated MWCNTs@AGE may also form significant spherical conductive defects in the epoxy resin. The AC resistivity, dielectric loss, and dielectric constant test data in Figure 4 also indirectly reflect the severe agglomeration of nanotubes inside the composite at high filler ratios.

Figure 3c,d show the SEM images of the brittle fracture of the epoxy resin composite with 20 wt.% BNNSs and 0.5 wt.% MWCNTs@AGE after liquid nitrogen treatment. Most of the BNNSs were embedded in the epoxy resin matrix and directly visible as they were lapped together to form thermal conductivity channels. In addition, although the BNNSs were more numerous and lapped together, no agglomeration occurred. The cross-sectional state of the nanotubes was also ideal, and some brittle cross-sections of MWCNTs@AGE can be seen on a flat section in the lower right corner of Figure 3c. They were evenly spaced and scattered throughout the field of view without severe agglomeration. Using SEM to observe the cross-section, it can be seen that the MWCNTs@AGE were dispersed between the BNNSs, connecting the independent BNNSs in series, and the two elements played a synergistic role in phonon transport in the heat conduction network. Furthermore, white particles can be observed in the figure, dispersed throughout the field of view, representing the added nano-SiO_2_ nanoparticles. When uniformly dispersed in the substrate, these nanoparticles could also enhance the overall thermal conductivity of the medium.

Insulation materials need not only high thermal conductivity to improve the thermal aging performance but also excellent dielectric properties. Figure 4a shows the variation of the resistivity of BNNSs/MWCNTs/SiO_2_ novel epoxy composites with the content of MWCNTs@AGE filler. Due to the excellent electrical conductivity and high aspect ratio of MWCNTs@AGE, providing channels for electron transfer, the AC resistivity of the composites showed a negative correlation with the filler mass fraction. With the exception of the low AC resistivity at 1 wt.% MWCNTs@AGE filler ratio, the AC resistivity of the remaining samples was on the order of 10^11^ at the working frequency, thus meeting the requirements of high–quality insulating materials.

Insulation materials need not only high thermal conductivity to improve the thermal aging performance but also excellent dielectric properties. Figure 4a shows the variation of the resistivity of BNNSs/MWCNTs/SiO_2_ epoxy composites with the content of MWCNTs@AGE filler. The MWCNTs@AGE with high electrical conductivity and high aspect ratio, making an easy establishing of electron transport channels in composites. As a result, the resistivity of the composites showed a negative correlation with the filler mass fraction. However, at 1 wt.% MWCNTs@AGE filler, the resistivity of the composites still higher than 10^11^ Ω·cm, thus meeting the requirements of high-quality insulating materials. In addition, the introduction of BNNSs, MWCNTs@AGE, and SiO_2_@KH560 also play a significant effect on the dielectric properties of the composites, as present in Figure 4b–f and Table 1. The dielectric constant of composites with only 0.5 wt.% SiO_2_ was 4.37 (at 1 kHz), which was significantly lower than that of pure epoxy (Figure 4b). While the introduction of BNNSs led to an increasing trend in the dielectric constant of the epoxy composites. At 20 wt.% BNNSs loading, the dielectric constant of the composites greatly increased to ~6.58 (1 kHz), a value of ~50.57% higher than that of pure epoxy. This can be attributed to the fact that BNNSs introduced a large number interfaces, contributing to a large amount of free charge was trapped, resulting in charge aggregation in the local space, i.e., interfacial polarization. A higher filler ratio of BNNSs resulted in a greater introduction of interfacial polarization, thus enhancing the overall polarization and increasing the dielectric constant [39,40,41]. In addition, in the composite system, the BNNSs in the form of flakes were dispersed and stacked together, which could form a capacitor-like structure. The micron-sized nanosheets acted as the pole plates of the capacitor, while the epoxy resin between the BNNSs acted as the dielectric of the capacitor. As the filler ratio increased, the distance between fillers decreased rapidly, and a higher filler ratio led to a faster decrease in distance. The density of the micro capacitors increased rapidly, thus explaining the faster increase in dielectric constant at a high filler ratio. In addition, the polar nature of BNNSs may have resulted in deflection vibration under the effect of the alternating electric field. Hence, their adjunction also enhanced the dielectric constant of the composite.

Compared to BNNSs, the addition of electrically conductive MWCNTs@AGE contributing to a higher dielectric constants for composites. When fixed the loading of SiO_2_ and BNNSs at 0.5 wt.% and 10 wt.%, respectively, the BNNSs/MWCNTs/SiO_2_/epoxy composite represent a high dielectric constant of ~5.68 at 0.5 wt.% MWCNT loading. The increase in the dielectric constant of the samples with a small amount of MWCNTs was tiny, while the increase in the dielectric constant was caused by a high concentration of fillers. This may have been due to the better agglomeration of MWCNTs after grafting AGE groups with minor van der Waals forces between AGE groups. Moreover, MWCNTs@AGE were subjected to high-power sonication for an extended duration before preparation and participation in epoxy resin curing, which unclustered the agglomerates and removed the longer nanotubes, reducing their aspect ratio. This led to an increase in spacing and a more uniform distribution among the MWCNTs@AGE, which were initially prone to agglomeration and entanglement. The introduction of a certain percentage of SiO_2_ nanoparticles also weakened the increase in dielectric constant brought by the addition of MWCNTs@AGE. This is because the dielectric relaxation frequency of dipole polarization and interfacial polarization inside the material was minor; therefore, when the frequency of electric field transformation increased, dipole polarization and interfacial polarization often did not occur in time, weakening the degree of polarization. Accordingly, a higher frequency resulted in a lower dielectric constant of the composite material. Under the action of the alternating electric field, the dielectric suffered energy loss due to both conductivity and polarization. The dielectric loss decreased slightly with increasing frequency and then increased with further increasing frequency. Due to the accelerated movement of polar molecules inside the material, the friction between molecular chains generated heat, which led to an increase in the dielectric loss of the material.

Furthermore, the higher conductivity and leakage current of MWCNTs led to an increase in the dielectric loss after filling with epoxy resin. As can be seen in Figure 4f, with the increase in the filler ratio of MWCNTs, the dielectric loss also showed a rapid increase. When the filler ratio exceeded 0.75 wt.%, the dielectric loss of the sample jumped, but the filler ratio of 1 wt.% also produced dielectric loss at an acceptable level. However, to reduce the dielectric loss of the insulation layer so as to reduce the power loss and insulation heating, the filler ratio of MWCNTs@AGE should be controlled. According to the experimental results, 0.5 wt.% is a relatively ideal ratio. In addition, to achieve the purpose of reducing the dielectric loss, some other fillers need to be added to reduce the dielectric loss of the composite. The test results show (Figure 4d) that the SiO_2_/epoxy obtained by adding a certain mass fraction of SiO_2_ nanoparticles to the pure epoxy resin significantly reduced the dielectric loss compared with the pure epoxy resin. The specific mass fraction of SiO_2_ nanoparticles was determined by several experiments. The KH560 used in the preparation of SiO_2_ nanoparticles introduced epoxy groups on the surface of SiO_2_ nanoparticles, which could participate in the curing of epoxy resin, thus tightening the bond between SiO_2_ nanoparticles and the epoxy resin substrate, weakening the charge aggregation at the interface, and reducing the polarization range. The reason for the decrease in dielectric loss may be that epoxy@SiO_2_ was able to trap electrons more efficiently; thus, the internal collisions between electrons and polymer macromolecules during the action of the alternating electric field were reduced, and the deeper traps increased the energy required to generate polarized charges, thereby reducing their generation. Additionally, the groups inserted onto the surface of nano-SiO_2_ were covalently bonded to the polymer macromolecules, which reduced the deflection vibration of the polar macromolecules in the composite material in the alternating electric field and weakened its polarization degree.

Stronger interphase interactions limit the migration and accumulation of space charges in nanocomposites. The synergistic effect of the two reduces the overall dielectric loss [42,43,44]. In fact, after adding nano-SiO_2_ nanoparticles to the epoxy resin with MWCNTs, the increase in dielectric loss tangent angle Tanδ was not significant compared with that of pure epoxy (Figure 4d), which shows that a certain content of nano-SiO_2_ nanoparticles had a considerable effect on the reduction in dielectric loss of the modified material and offset the high dielectric loss brought by MWCNTs@AGE.

However, BNNSs led to a massive enhancement in thermal conductivity while also causing an increase in dielectric loss (Figure 4e). Overall, their introduction led to a slight enhancement; at 0.5 wt.% MWCNTs@AGE and SiO_2_@KH560, with a 20 wt.% BNNSs filler ratio, the maximum value of dielectric loss in the low-frequency band was about 0.05 (i.e., >0.03). However, its Tanδ value in the whole frequency range was still relatively low. The reason for BNNSs increasing the Tanδ of epoxy resin composites is similar to the reason for the increase in dielectric constant proposed in the previous section, i.e., the nanosheet filler gradually decreased the trap depth inside the material, necessitating less energy to generate an equal amount of polarized charge. The degree of interfacial polarization was significantly increased, thus increasing the polarization loss. The steering polarization caused by the tiny vibrations resulting from the nanosheets’ polarity also caused an increase in the dielectric loss. In addition, the free electrons, particles, and atoms introduced by the filler produced a resonance effect during vibration, introducing resonance loss, which also increased the dielectric loss of the material. However, the material still presented a low level of dielectric loss overall [39].

**Figure 5:**
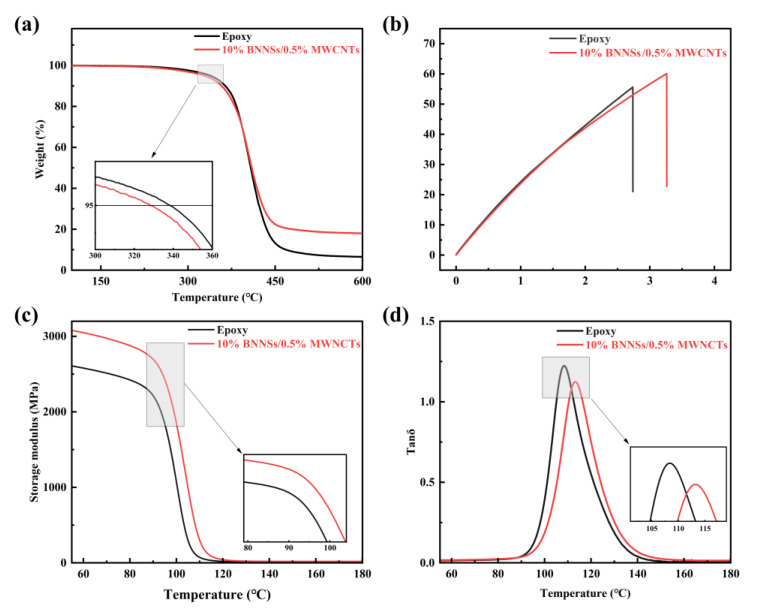
(**a**) TGA curves (all samples contain SiO_2_@KH560). (**b**) Stress–strain curves (All samples contain SiO_2_@KH560). (**c**,**d**) DMA curves.

Figure 5a shows the thermogravimetric analysis (TGA) results of composites. The initial thermal decomposition temperature T_5%_ is the temperature corresponding to when the mass loss of the material reached 5%. We can see that the T_5%_ of pure epoxy resin was 338.35 °C, However, the T_5%_ of the 10 wt.% BNNSs and 0.5% MWCNTs sample decreased to 328.39 °C, because the BNNSs filler did not form a covalent bond with the matrix, thus reducing the crosslinking of the polymer around the fillers. However, the thermal stability of the composite is not greatly affected.

According to the tensile test of dumbbell–shaped specimens, we found that the toughness of the material was significantly improved after adding nanotubes, as mainly reflected in the slope of the stress–strain curves (Figure 5b), which remained unchanged, while the maximum deformation degree was significantly improved.

We obtained the Tanδ (Figure 5d) and energy storage modulus (Figure 5c) of the composite through dynamic thermomechanical analysis (DMA). The glass transition temperature of the modified composites changed, and the addition of BNNSs with a low percentage of MWCNTs@AGE increased the glass transition temperature. This may be due to the fact that BNNSs and MWCNTs@AGE restricted the space for the free movement of epoxy molecular chains. Interestingly, when the filler ratio of MWNCTs@AGE increased, the glass transition temperature increased and then decreased, while the magnitude of energy storage loss appeared to decrease and then increase. This was very similar to the trend of thermal and dielectric conductivity. Both observations were likely due to the fact that nanotubes could be well dispersed in the composite at lower ratios to exert their modification effect and enhance the degree of crosslinking, which in turn reduced the friction between the molecular chain segments and the energy loss. However, with increasing filler content, MWCNTs@AGE agglomerated and a large number of defects were introduced, which reduced the degree of crosslinking and increased the loss [45].

The filler ratio of MWCNTs@AGE and the numerical magnitude of the energy storage modulus of the material showed a positive correlation, due to the fact that energy storage represents rigidity, and the increase in crosslink density increased the energy storage modulus and strengthened the mechanical properties. This indicates that the addition of nanotubes had an enhancing effect on the mechanical properties of the composites. This was due to the longer structure of nanotubes interspersed in the resin matrix and the presence of a large number of binding sites with functionalized groups on the surface crosslinked to the resin, which effectively restricted the movement of the epoxy resin chains with a large number of BNNSs fillers [45,46]. Thus, the cracks frequently turned at the interface between the reinforcement and the matrix, forming many small, curved surfaces, resulting in a rough cross-section of the composite. In Figure 3c, we can clearly observe substantial distortion in the cross-section, indicating that the MWCNTs@AGE filler played its role in enhancing the mechanical properties.

## 4. Conclusions

In conclusion, the surface functionalization of MWCNTs with AGE introduced functionalized groups to enhance the interfacial interphase bonding between MWCNTs and epoxy. XPS, FT–IR, and Raman spectra confirmed that the AGE groups were successfully grafted onto the surface of nanotubes. The thermal conductivity of the BNNSs/MWCNTs/SiO_2_@KH560/epoxy was as high as 0.9327 W m^−1^ K^−1^, resulting in the synergistic construction of thermal conductivity networks of different scales and dimensions between MWCNTs and BNNSs. The functional groups introduced by AGE resulted in a tighter covalent bond between the nanotube and the resin substrate, which not only reduced the interfacial thermal resistance but also improved the dynamic mechanical properties of the composite material and enhanced the toughness and resistance of the material. In addition, SiO_2_@KH560 introduced deeper traps, which reduced the dielectric loss level and improved the crosslinking degree of the material. We believe that the results of this study provide a facile method for preparing insulating materials with excellent dielectric properties, high thermal conductivity, and crack resistance.

## Figures and Tables

**Table 1:** Volume resistivity and dielectric properties of composites at 1000 Hz.

Composites	Dielectric Constant	Dielectric Loss	Volume Resistivity (Ω cm)
Epoxy	5.02	0.0047	1.07819 × 10^11^
0.5 wt.% SiO_2_	4.37	0.0039	1.28016 × 10^11^
1% BNNS + 0.5 wt.% SiO_2_	4.61	0.0069	1.21 × 10^10^
5% BNNS + 0.5 wt.% SiO_2_	4.99	0.0070	3.84 × 10^10^
10% BNNS + 0.5 wt.% SiO_2_	5.13	0.0075	3.95 × 10^10^
15% BNNS + 0.5 wt.% SiO_2_	5.34	0.0141	4.44 × 10^10^
20 % BNNS + 0.5 wt.% SiO_2_	5.67	0.0148	7.75 × 10^10^
0.125% MWCNTs (10 wt.% BNNS + 0.5 wt% SiO_2_)	4.62	0.00632	1.07815 × 10^11^
0.25% MWCNTs (10 wt.% BNNS + 0.5 wt% SiO_2_)	4.91	0.0078	9.4142 × 10^11^
0.5% MWCNTs (10 wt.% BNNS + 0.5 wt% SiO_2_)	5.68	0.0085	2.77624 × 10^10^
0.75% MWCNT (10 wt.% BNNS + 0.5 wt% SiO_2_)	6.14	0.0174	2.61256 × 10^10^
1% MWCNTs (10 wt.% BNNS + 0.5 wt% SiO_2_)	6.58	0.0247	4.21098 × 10^9^

## Data Availability

The data presented in this study are available on request from the corresponding author.
